# Analysis of the LNAPL Migration Process in the Vadose Zone under Two Different Media Conditions

**DOI:** 10.3390/ijerph182111073

**Published:** 2021-10-21

**Authors:** Rui Zuo, Xiao Zhao, Jie Yang, Minghao Pan, Zhenkun Xue, Xiang Gao, Jinsheng Wang, Yanguo Teng

**Affiliations:** 1 College of Water Science, Beijing Normal University, Beijing 100875, China; zr@bnu.edu.cn (R.Z.); 201921470033@mail.bnu.edu.cn (X.Z.); 202021470021@mail.bnu.edu.cn (M.P.); xzkbjsf@163.com (Z.X.); donghuixu123@126.com (X.G.); wangjs@bnu.edu.cn (J.W.); teng1974@163.com (Y.T.); 2 Engineering Research Center of Groundwater Pollution Control and Remediation, Ministry of Education, Beijing 100875, China

**Keywords:** LNAPL, transport, vadose zone, petroleum hydrocarbon pollutants, groundwater

## Abstract

This study focused on the processes of free infiltration, precipitation displacement, and natural attenuation of the LNAPL under the condition of near-surface leakage. Sandbox experiments were performed to explore the migration characteristics of LNAPL in the vadose zone with two media structures and the influences of the soil interface on the migration of LNAPL. The results indicate that the vertical migration velocity of the LNAPL infiltration front in medium and coarse sand was 1 order of magnitude higher than that in fine sand and that the LNAPL accumulated at the coarse–fine interface, which acted as the capillary barrier. Displacement of precipitation for LNAPL had little relationship with rainfall intensity and was obviously affected by medium particle size, where coarse sand (40.78%) > medium sand (20.5%) > fine sand (10%). The natural attenuation rate of the LNAPL in the vadose zone was related to the water content of the media; the natural attenuation rate of fine sand was higher. This study simulated the process of the LNAPL leakage from the near surface into the layered heterogeneous stratum, improved the understanding of the migration of the LNAPL under different stratum conditions, and can provide support for the treatment of LNAPL leakage events in the actual site.

## 1. Introduction

The pollution of groundwater and soil by nonaqueous phase liquids (NAPLs) occurs frequently [1,2,3], which has always aroused great concern in environmental preservation [3,4,5]. Because LNAPLs are not miscible with water, after they enter the subsurface, air, water, and NAPLs within the porous media can form a multiphase flow system [6], and the migration characteristics become more complex and difficult to study in contrast with those of usual solute migration [7].

When NAPLs are released into the subsurface, they first migrate within the vadose zone under the influence of gravitational hydrodynamic and capillary forces [8]. Numerous studies have indicated that NAPL migration in the subsurface is governed by complex mechanisms that are influenced by many factors [9], including permeability [10], porosity, pore-size distribution [11], and the surface properties of the porous media [12,13]. Some scholars have studied pore scale interactions on the transport and fate of organic pollutants [14,15].The migration paths of LNAPLs are uniform pore channels, so porosity and pore size exert a certain control on the migration and interception of LNAPLs. In addition, LNAPLs need to compete with water in pores for pore space, so the saturation of the media is also an important factor affecting the migration of LNAPLs [16]. Affected by complex conditions, there will be frozen water in the soil of some regions [17], such as the Qinghai-Tibet Plateau, which will affect the migration of pollutants, making it more complex to explore the mechanism of pollutant migration. This study does not consider such situations.

In addition to the above factors, precipitation as the initial external driver cannot be ignored for the migration of LNAPLs in the vadose zone. LNAPLs are moved downward by rainfall recharge. In addition, rainfall recharge can significantly reduce the residual LNAPL saturation in the upper part of the capillary interface [18,19]. Residual petroleum in the soil is desorbed and released under certain rainfall leaching conditions, thereby accelerating the migration of pollutants towards the saturated zone. Through soil column experiments, Frollini [20] studied soil media trapped with residual LNAPLs under rainfall leaching conditions. It was concluded that the volume of leaching water is exponentially correlated with the cumulative mass of dissolved-phase LNAPLs in effluent solutions. LNAPL displacement and entrapment occur during seasonal water table fluctuations, and LNAPL recovery is affected by precipitation [21,22,23]. With the leaching effect of precipitation, the petroleum contaminants trapped in the vadose zone and capillary zone further penetrate and contaminate groundwater.

Under natural conditions, the unsaturated zone is typified by many different structures; hence, it is not typically homogeneous [24,25]. The structure of the unsaturated zone affects the rate and pattern of liquid migration, and the capillary barrier exists at the fine–coarse interface [26]. Kechavarzi [27] conducted two two-dimensional multiphase flow experiments to investigate the effect of textural interfaces on water and LNAPL pressure and saturation distribution. The results show an important capillary barrier effect on water and LNAPL flow at the interface of fine sand layers overlaying coarse sand deposits. Schroth [28] simulated the migration of LNAPLs in an inclined two-dimensional heterogeneous sand tank and found that the capillary difference of the media interface formed an obstacle, while the migration of LNAPLs was largely influenced by the water saturation of the fine sand above the heterogeneous interface. Wipfler [29] studied the migration process of LNAPLs in heterogeneous strata by sand tank experiments and concluded that the difference in capillary interaction between different sand layers is the most dominant controlling factor in determining the distribution of LNAPLs. Therefore, the stratification of the media can influence the migration of LNAPL, but how to affect it needs to be further explored.

This study selected diesel as a typical LNAPL, and based on two-dimensional sand tank simulation experiments with different media filling, the purpose was to explore the migration and distribution characteristics of LNAPLs in the vadose zone, as well as the water oil displacement driven by precipitation and natural attenuation process. In addition, the migration characteristics of LNAPL from coarse to fine media and from fine to coarse media were explored.

## 2. Materials and Methods

### 2.1. Two-Dimensional Migration Equipment

**Figure 1:**
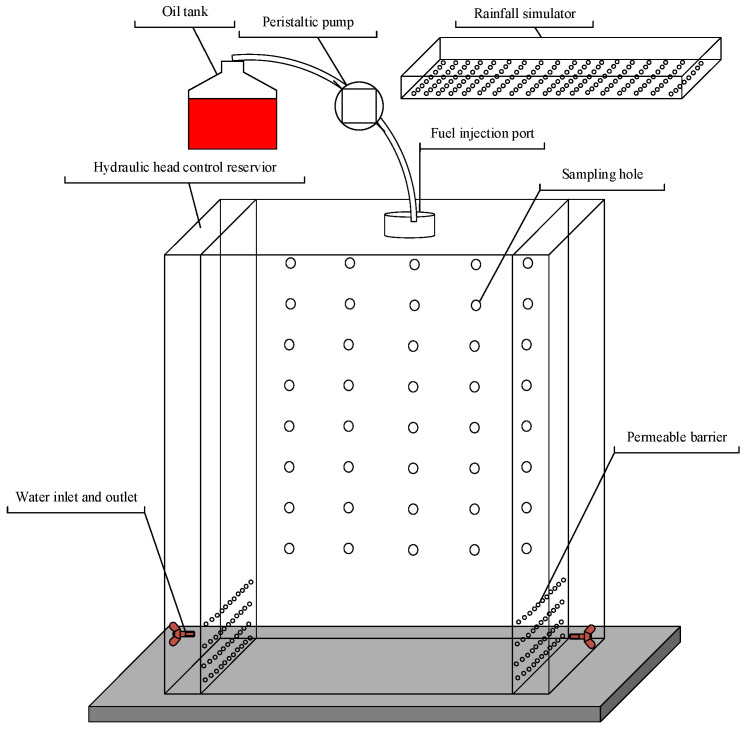
Schematic representation of the flow tank and rainfall simulator.

Tests were carried out in a two-dimensional flow tank with dimensions of 70 cm long × 100 cm high × 5 cm thick (Figure 1) to simulate the infiltration process of LNAPLs in the vadose zone, and the infiltration front of dyed LNAPLs was recorded by a digital camera. The tank body was divided into three parts: there were two hydraulic head control reservoirs that were 5 cm wide connected on both sides of the tank, and for each reservoir, there were ports that were used to control the hydraulic head at each of the boundaries. The main experimental area with a width of 60 cm in the middle was filled with soil media. The top was designed with the fuel injection port connected to the peristaltic pump; the peristaltic pump was used to control diesel release rates. There was a permeable partition between hydraulic head control reservoirs and the main experimental area, which was made of a double-layer plexiglass screen plate sandwiched with three-layer stainless steel mesh to ensure a hydraulic connection and prevent the migration of fines from the soil media into the hydraulic head reservoir. The front of the tank was a smooth and transparent observation surface. On the back of the tank (Figure 1), there were 8 rows and 5 columns, with 40 sampling apertures installed in total; the sampling apertures were plugged with rubber plugs. 

### 2.2. Media and Materials

The contaminated site was a gas station that had been abandoned for ~20 years. There were six underground diesel oil storage tanks at the site. The bottoms of the oil tanks were not designed to prevent seepage, and the surface soil was bare. The soil used in this experiment was collected from approximately 5 m below the ground surface; the media of this layer was medium sand and coarse sand. After natural air-drying for 48 h, animal and plant residues and large pebbles (with diameters greater than 4 cm) were removed. Soil particles were sorted by using sieves of different mesh numbers. Coarse sand, medium sand, and fine sand were selected as the representative filling media. The specific gravity of the media (Table 1) was measured by the gravimetric method.

The LNAPL that was used in the experiments was diesel. Diesel has various uses and is a common oil product that is widely available and used. It is quite probable that diesel is released into the environment during normal storage and use practices, so using diesel in the experiments was relevant to real situations [30,31]. Diesel is less dense than water and is also immiscible in water, making it a typical LNAPL. The relevant physical properties of diesel used in this experiment are shown in Table 2.

### 2.3. Experiment Designs

#### 2.3.1. Configurations of the Porous Media

**Figure 2:**
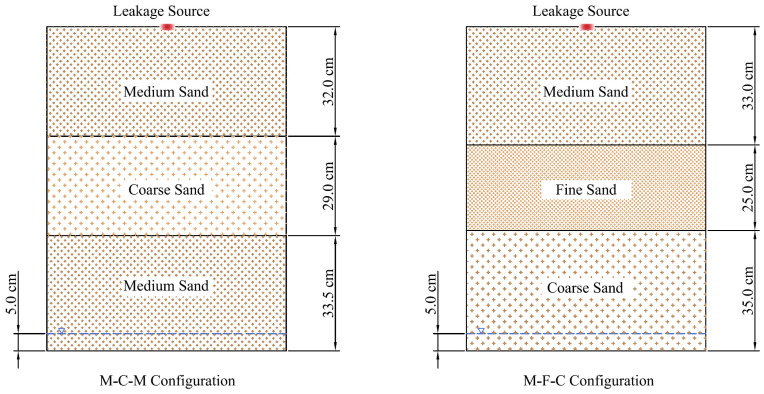
Schematic diagram of soil media structures.

Two types of media configurations were designed (Table 3). During the filling process, the media layer was compacted every 3 cm to ensure that the height of each layer reached the designated height. Prior to placing and compacting each sand layer, the surface of the previously compacted layer was scarified or raked to ensure homogeneity at the interface of the layers. Then, the porosity of the filling soil media was calculated according to the bulk density and specific gravity of the soil. The eventual filling configuration is shown in Figure 2. According to the media filling structures, the two sand tanks were named M-C-M and M-F-C (M referred to medium sand, C referred to coarse sand, F referred to fine sand).

#### 2.3.2. Experimental Procedure

At the beginning of the experiment, the water was injected from the bottom inlet, and the water level was gradually raised. When the top of the sand tank was completely saturated, the bottom outlet was opened after standing for 24 h so that the water in the sand tank could be drained naturally by gravity until a water table was reached at 5 cm above the bottom of the tank. During the next 12 h, no outflow of water was measured, and water was considered at steady state and at hydrostatic pressure.

##### Diesel Injection Experiment

Prior to diesel injection, stratified sampling from the sand tank was performed to determine the water content, which was determined via the drying method in an oven at 110 °C for 8 h. The saturation of soil water was further calculated according to the specific gravity and bulk density of the soil. To ensure the visibility of diesel during the experiment, the diesel was dyed with Sudan III with a mass ratio of 0.01%, which gave the diesel fuel a bright red colour for easy visualization and image interpretation. A video camera on the front of the sand tank was used to capture images and record the experimental process, and a marker was used to plot the spill range of the dyed diesel at different moments during the experiment. A peristaltic pump and the fuel injection port were used to leak the dyed diesel over the top of the sand tank. The diesel leakage time and leakage volume are shown in Table 4.

The dyed diesel was bright red, which had a strong contrast with the colour of the soil medium. On this basis, the movement process of the diesel front was recorded by a video camera. The images at different times were processed with Photoshop CS6 Chinese version [32].

##### Precipitation Leaching Experiment

The migration of diesel in the sand tank was observed. After 96 h of diesel release, its distribution in the sand box was relatively stable, at which time the LNAPL was in an equilibrium retention state under the interaction of gravity and capillary force in the vadose zone. Prior to the precipitation leaching experiment, soil samples were collected through the sampling apertures with a sample spoon on the back of the sand tank. The total petroleum hydrocarbons (TPH) concentration in the samples were detected by infrared oil detector.

Precipitation leaching experiments were conducted for the M-C-M and M-F-C tanks with various precipitation features. Rainfall was simulated by leakage from the inner holes of simulator (Figure 1). The rainfall simulator was placed on the top of the sand tank. The designed precipitation intensity, precipitation duration, and total precipitation volume of the M-C-M tank were 250 mL/min, 12 min, and 3000 mL, respectively, and the designed precipitation intensity, precipitation duration and total precipitation volume of the M-F-C tank were 2000 mL/min, 1 min, and 2000 mL, respectively. 

During the precipitation experiment, the bottom outlet was kept open to ensure that the water table did not rise with precipitation and to maintain the unsaturated state of the unsaturated zone. After the precipitation experiment, the bottom outlet was kept for 24 h until there was no more liquid flowing out of it. Soil samples through the sampling apertures were collected again, and the total petroleum hydrocarbons (TPH) concentration in the samples was detected by infrared oil detector.

The sampling monitoring method can more truly reflect the change of concentration at each point in the sand tank [33]. In this study, the change of TPH at the same point before and after rainfall was used to characterize the displacement effect of precipitation on diesel; the displacement rate of diesel by rainfall was obtained by dividing the TPH difference before and after rainfall by the TPH before rainfall.

##### Natural Attenuation Process Monitoring

The water table in both tanks was maintained at 5 cm when the precipitation experiments were completed. In addition, the tops of the tanks were kept open to simulate the natural volatilization process. Both tanks were continuously monitored for 6 months, and soil samples were collected from different layers for TPH determination every 30 days.

The changes of TPH in the whole sand box were reflected by the changes in the average TPH at each point in the sand box, at the same time, by comparing the changes in LNAPL content at different depths to explore the factors affecting the natural attenuation.

## 3. Results

### 3.1. Free Migration of the LNAPL

#### 3.1.1. Free Migration of the LNAPL in the M-C-M Tank

**Figure 3:**
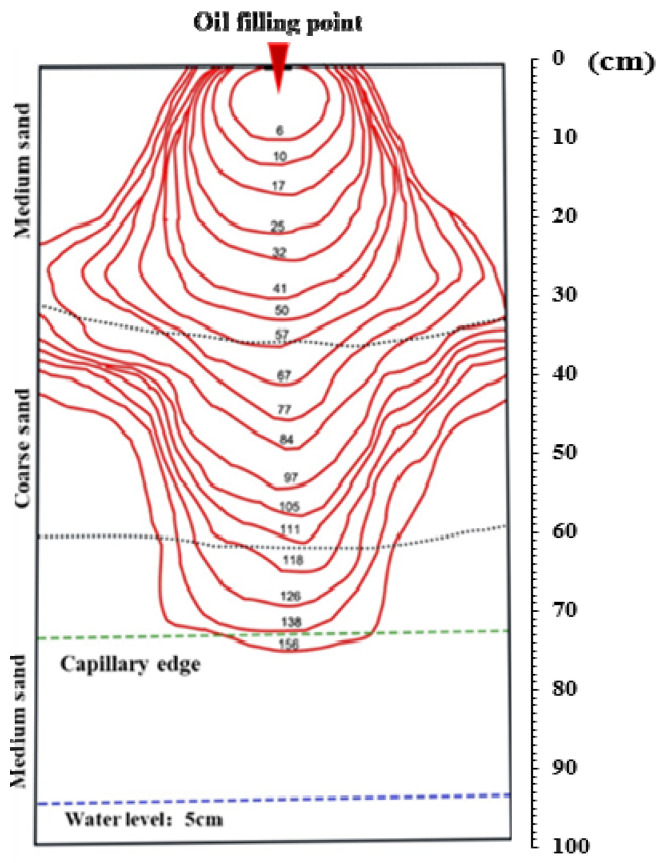
Distribution of the LNAPL infiltration front at different times in the M-C-M tank.

Based on the captured images, the distribution images of LNAPL at different times were processed by Photoshop, and the Figure 3 describes the LNAPL free migration process that were obtained. At the initial stage of infiltration, the LNAPL migrated rapidly in the homogeneous medium in an approximately circular shape, and the oil peak in the sand tank showed a finger flow phenomenon, which was due to the microscopic heterogeneity of the medium, which resulted in different seepage rates in some regions. The vertical and horizontal migration rates were similar, which indicated that the capillary force on the LNAPL was more obvious in the near-surface of infiltration. The LNAPL continuously migrated under the force of capillary and gravity. When the infiltration front of diesel reached the interface between medium sand and coarse sand, the lateral migration was obviously intensified, and the shape of the LNAPL pollution body also changed from approximately circular to gyroscopic, while the infiltration front reached the sidewall of the sand tank. After the infiltration front broke through the interface between medium sand and coarse sand, gravity became the main driver in the migration process of LNAPLs, and the vertical migration degree was greater than the horizontal migration degree. As LNAPLs continued to infiltrate downward, the lateral diffusion controlled by the capillary force of the soil medium gradually decreased, while the vertical infiltration driven by the gravity force of the LNAPLs continued.

Since the capillary pressure of medium sand was greater than that of coarse sand, when the LNAPL migrated to the medium–coarse interface, the oil pressure could not reach the entry pressure of the medium sand layer, so accumulation occurred at the interface. LNAPL spread laterally along the upper medium–coarse interface. This interface apparently acted as a capillary barrier to the LNAPL [29]. Due to this accumulation, the capillary pressure between the water and the NAPL phase was decreased and eventually declined to a value where the (now non-wetting) LNAPL relative permeability of the coarse layer was greater than in the medium layer. Then, LNAPL infiltrated into the coarse layer.

#### 3.1.2. Free Migration of the LNAPL in the M-F-C Tank

**Figure 4:**
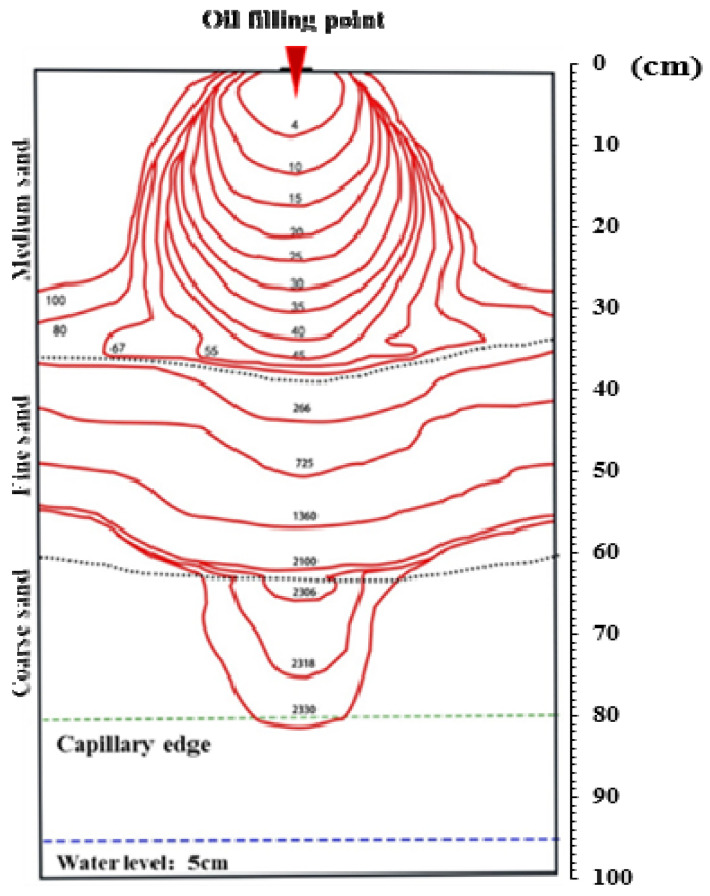
Distribution of the LNAPL infiltration front at different times in the M-F-C tank.

The migration of the LNAPL in the initial infiltration in the M-F-C tank was similar to that in the M-C-M tank (Figure 4): it was nearly circular and rapidly transported in homogeneous medium sand. When the oil peak approached the soil interface of medium sand and fine sand, the vertical infiltration almost stopped, and the LNAPL accumulated at the soil interface and spread to both sides of the sand tank. As the LNAPL gradually accumulated, the migration front broke through the limitation of the lithologic interface and continued vertically at 266 min. The LNAPL showed piston infiltration in the fine sand layer at a very slow speed; it took approximately 2300 min to penetrate the fine sand layer and reach the interface between fine sand and coarse sand. The LNAPL, which first arrived at the coarse sand, formed a new oil front, gradually expanded, and rapidly infiltrated the capillary water surface for approximately 2330 min.

Capillary forces played an important role in the case of multi-phase fluid flow in layered heterogeneous systems. Fine sand had a higher entry pressure than medium sand because its permeability was less than that of medium sand; consequently, if the LNAPL entered a fine sand layer, the capillary pressure needed to reach a higher level. Prior to entry, the LNAPL accumulated and spread above the interface between medium and fine sand. 

#### 3.1.3. Analysis of Influencing Factors of LNAPL Migration under Different Media Structures

**Figure 5:**
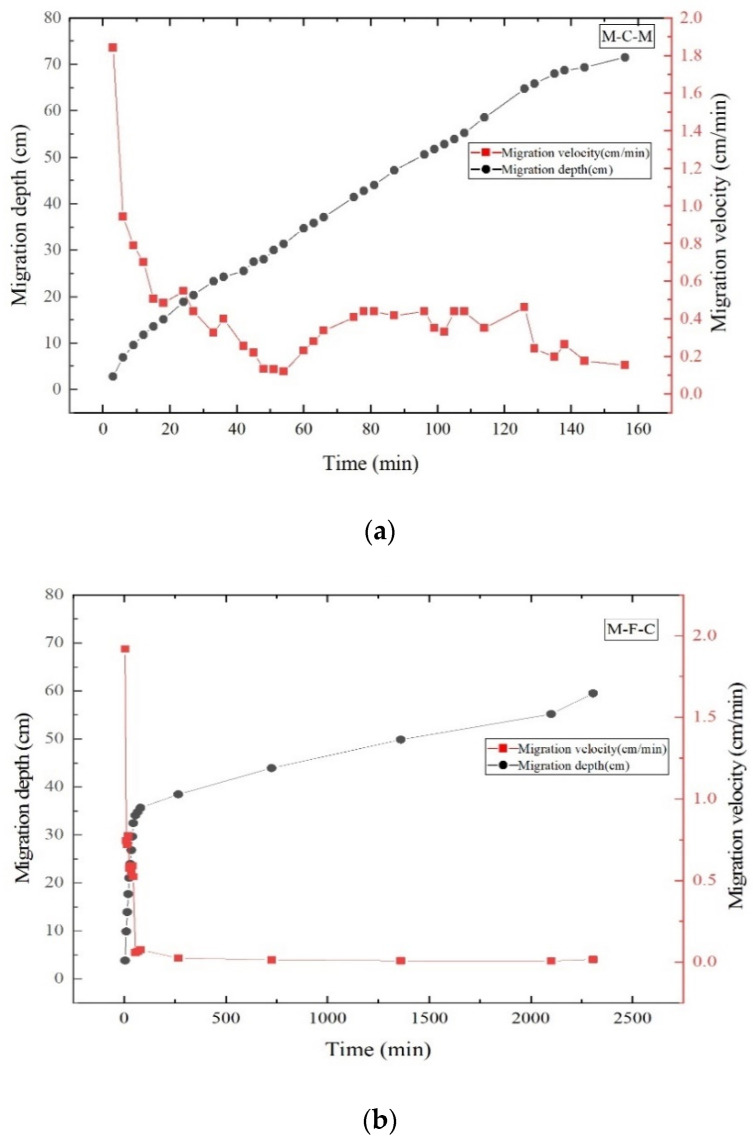
Variation of longitudinal migration depth and migration rate of LNAPL with time in the M-C-M tank (**a**) and M-F-C tank (**b**) (before entering the third layer of media).

It was of great significance to explore the vertical migration characteristics of the LNAPL for the near-surface leakage events because the infiltration process of the LNAPL was controlled by formation conditions. In two different cases (from relatively coarse-grained media to relatively fine-grained media and from relatively fine-grained media to relatively coarse-grained media), the vertical migration characteristics of LNAPL showed obvious differences (Figure 5). The vertical migration velocity of LNAPL in the upper medium sand layer was similar in the two sand tanks. In the M-C-M sand, the vertical migration velocity of LNAPL in the middle coarse sand layer was about 0.3 cm/min, while in the M-F-C tank, the vertical migration velocity of LNAPL in middle fine sand layer was about 0.025 cm/min. In the two sand tanks, the longitudinal migration velocity of LNAPL in the interlayer media was different by an amount. In the M-C-M sand tank, it took only 110 min for the diesel migration front to reach the depth of 60 cm (that is, near the of the second medium interface), while in the M-F-C sand tank, it took 2100 min for the diesel migration front to reach the depth of 60 cm. In this process, fine sand was the key control layer, which determined the overall migration rate.

**Figure 6:**
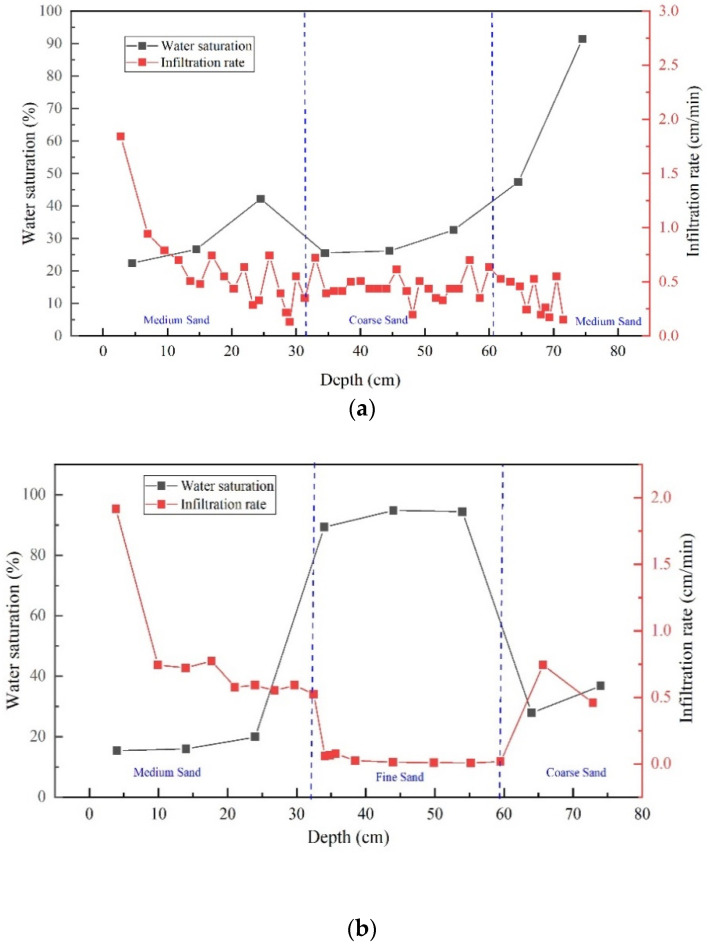
Comparison between water saturation and infiltration rate of the diesel front: (**a**) M-C-M; (**b**) M-F-C.

The infiltration rate of LNAPLs was associated with soil properties. Gavin [34] and Yang [35], through a soil column simulation experiment of rainfall infiltration in an unsaturated zone, found that the migration rate of the wet front of water-infiltrated soil was closely related to soil saturated permeability. Wang [36] demonstrated through oil infiltration column experiments that the oil infiltration rate and the migration rate of the migration wet front decreased exponentially, and the progression of the wetting front and crude oil was significantly affected by the size of the pore space. When non-wetting fluid entered the media containing wetting fluid, the influence of media saturation was very important. Here, there was a relationship between the front infiltration rate of the LNAPL and the saturation of media (Figure 6). The infiltration rate (cm/min) at different times could be obtained by cutting the infiltration image of the oil peak every 3 min. The infiltration rate was compared with the water saturation of different infiltration depths. The migration rate of the LNAPL in two tanks was related to the water saturation: the higher the water saturation was, the lower the migration rate of the LNAPL front.

High water saturation mean that soil pores were occupied by water, resulting in a decrease in the relative permeability of the LNAPL. Relative permeability depended on fluid saturation. The LNAPL front migration rate in the fine sand layer was very slow and almost stagnant because of the high water saturation of the fine media.

### 3.2. The Driving Migration Process of the LNAPL 

#### 3.2.1. The Driving Migration Process of the LNAPL in the M-C-M Tank

**Figure 7:**
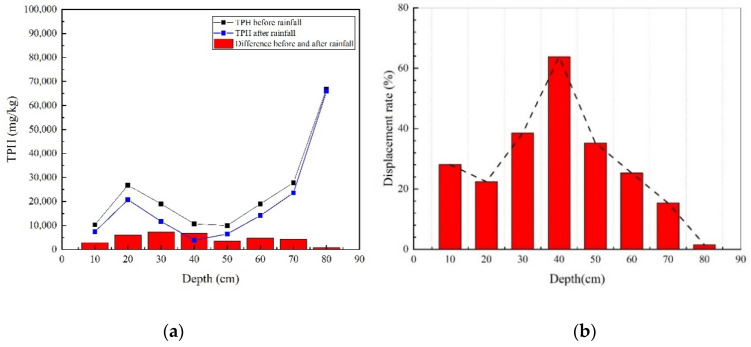
(**a**) TPH concentration before and after rainfall and (**b**) diesel displacement rates at different depths in the M-C-M tank.

At the end of the LNAPL injection stage, the LNAPL reached a state of equilibrium whereby the capillary and gravitational forces balanced out each other. This equilibrium status changed once rainfall infiltrated the contaminated media. Precipitation can drive the redistribution of LNAPL in the vadose zone to continue to move. By sampling and analysing the TPH at the same point before and after precipitation, the variation of TPH at different depth points before and after rainfall could be obtained (Figure 7a); it can be seen that the TPH of each point after rainfall was less than that before rainfall, which confirmed that rainfall will drive LNAPL to continue to migrate downward. At the interface between medium and coarse media, the depth was about 30 cm, and the TPH difference before and after rainfall was the largest. Whether before or after rainfall, the TPH value at the depth of 80 cm was the highest, which showed that LNAPL was gathered here, while in the middle coarse sand layer, the TPH value was the lowest, which meant that the interception of LNAPL by the coarse sand layer was the least.

The percentage of the difference relative to the TPH before precipitation was the displacement rate of precipitation on the LNAPL at that point (Figure 7b). The highest displacement rate was 64% at a depth of 40 cm. The displacement rate of medium sand in the upper and lower medium sand layers was lower than that of the middle coarse layer, and the removal rate of the upper and lower medium sand sections was not higher than 30%. The reason was that the permeability of coarse sand was higher than that of medium sand. Additionally, the local hydraulic gradient during rainfall was larger than that of medium sand, and the capillary force between coarse sand particles was smaller than that of medium sand; thus, the NAPL phase in the coarse sand medium more easily eluted into the flowing state. 

#### 3.2.2. The Driving Migration Process of the LNAPL in the M-F-C Tank

**Figure 8:**
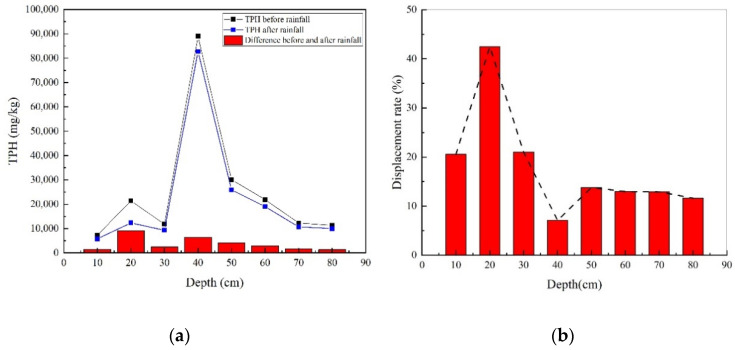
(**a**) TPH concentration before and after rainfall and (**b**) diesel displacement rates at different depths in the M-F-C tank.

Using the same method as in the previous section, the changes of TPH and displacement rates before and after precipitation at different depths in the M-F-C tank were obtained (Figure 8). As can be seen from Figure 8a, the value of TPH at the depth of 40 cm was the highest, indicating that more LNAPL had accumulated in the middle fine sand layer. Compared with the M-C-M tank, the interception capacity of the middle fine sand layer of the M-F-C tank for LNAPL was much greater than that of the middle coarse sand layer.

It can be seen that the changes of TPH before and after rainfall was smaller than that in the M-C-M tank. At the depth of 20 cm, the displacement of the LNAPL by precipitation was the largest. The possible reason was that this depth was affected by a large hydraulic pressure, resulting in a high removal rate. Near the depth of 40 cm, although TPH was much higher than other depths, there was no obvious difference in displacement; moreover, the displacement rate was the smallest. Because the capillary force of fine sand itself was large and the permeability was small, the pressure of water in the infiltration process was not enough to replace most of the NAPL phase. Finally, due to the capillary barrier effect of the interface between the medium and fine sand, when the water reached the fine sand layer, the speed slowed, and the removal rate was low.

#### 3.2.3. Analysis of Influencing Factors of Driving Migration Process under Different Media Structures

The designed precipitation intensity of the M-C-M tank was 250 mL/min, and the designed precipitation intensity of the M-F-C tank was 2000 mL/min. The two precipitation intensities did not have much effect on the displacement of the LNAPL.

**Figure 9:**
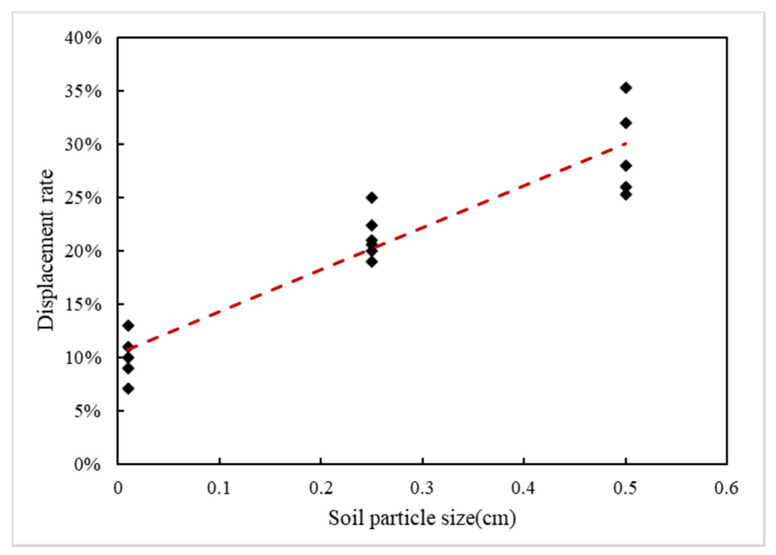
Relationship between the soil particle size and displacement rate.

In this study, we compared the relationship between precipitation displacement rate and medium particle size in different media. Figure 9 shows the relationship between soil particle size and the displacement rate. There was a slight positive correlation between the displacement rate and soil particle size. Kechavarzi [37] suggested in an experimental study that soil media with larger soil particle sizes have more void space and tend to hold more LNAPLs during the LNAPL infiltration process. In addition, the trapped free phase LNAPLs can be driven out of void space in the process of water flow displacement. In contrast, because the specific surface area of small particles was large and the pore throat between voids was small, LNAPL was not easy to be displaced by water flow. As a result, the free phase LNAPL was more likely to migrate downward in media with larger particles, while in media with smaller particles, LNAPL was more likely to be retained during the water flow displacement process. This was consistent with the viewpoint put forward by Frollini [20], who found that the denser the soil medium, the greater the residual amount of LNAPL. However, the dissolution amount was very small because the available pores that the water flow could pass through were very small.

In the process of driving the migration process, the influence of different media configurations on the LNAPL displacement rate was reflected in the lithology. Media with different lithologies had different particle sizes. The larger the particle was, the more easily the residual LNAPLs in the pores were displaced by water flow, and LNAPLs in the pores of the media with small particles was more likely to be retained.

### 3.3. Natural Attenuation Process of the LNAPL

#### 3.3.1. Natural Attenuation Characteristics of the LNAPL in the M-C-M Tank

**Figure 10:**
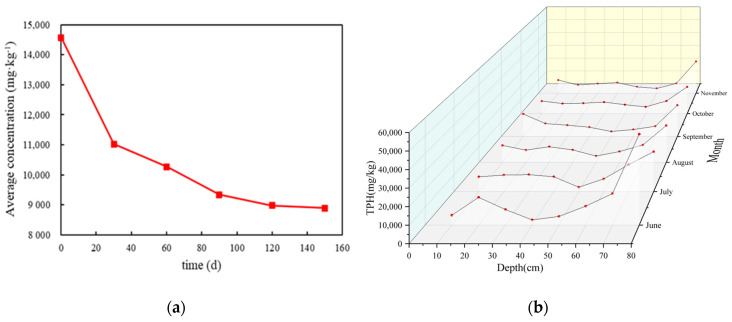
(**a**) Variation in the TPH average concentration and (**b**) monthly concentrations of different points in the M-C-M tank.

The sand tank was naturally placed for 150 days, and the changes of the total concentration of TPHs in the tank were used to reflect the natural attenuation of LNAPLs (Figure 10a). After 150 days, the average TPH concentration in the tank decreased to 61.01% of the initial value. In the initial stage, the TPH concentration decreased rapidly by 24.32% in 30 days, accounting for 62.37% of the total attenuation in 150 days. In the later stage, the decay rate of LNAPLs continued to decline.

From the comparison of TPH concentrations at different depths during each month in the M-C-M tank (Figure 10b), the TPH concentration in the coarse sand was less than that in the medium sand, which was related to soil structure and particle size. In addition, the TPH value near 80 cm was always at a high point. This was because after LNAPLs entered the phreatic surface, a thin "oil slick" layer was formed on the water surface. At the same time, the capillary pressure of the capillary zone also made some of the LNAPLs stay at the edge of the capillary zone, which made the soil more vulnerable to the influence of "residual oil" volatilization in groundwater, resulting in a high TPH value.

Overall, although the average TPH concentration in the whole tank gradually decreased with time, the TPH concentration at the same depth sometimes fluctuated with time, especially in the surface soil. Gidda [38] studied the volatilization behaviour of gasoline in aqueous media and found that gasoline would migrate to the surface of soil at a certain oil content; this phenomenon was called “wick” movement. Along with volatilization, the LNAPLs in the deep layer migrated upward in the form of steam, which made the TPH value in the sand fluctuate.

#### 3.3.2. Natural Attenuation Characteristics of the M-F-C Tank

**Figure 11:**
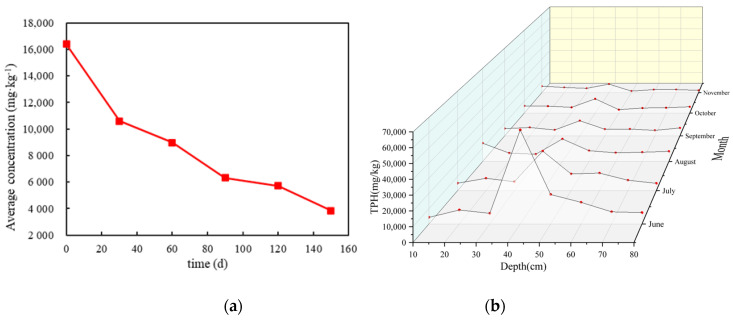
(**a**) Variation in the average concentration of TPHs and (**b**) monthly concentrations of different points in the M-F-C tank.

Similar to the M-C-M tank, the total TPH concentration in the M-F-C tank gradually decreased over time during natural placement (Figure 11a). After 150 days, the average TPH concentration in the tank decreased to 23.40% of the initial value—namely, the attenuation was 76.60%. Different from the M-C-M tank, the natural attenuation process of the M-F-C tank was relatively gentle, and the initial concentration decreased by 35.46% in the first 30 days, accounting for 46.30% of the total attenuation in 150 days. The loss rate of TPHs was faster in the early stage of attenuation, which was mainly due to the high concentration of pollutants in the early stage, and the concentration of volatile substances was also higher. According to the principle of volatilization, the volatilization rate was faster when the concentration was high, and the volatilization loss accounted for a large proportion of natural attenuation. When the concentration of volatile substances decreased to a certain extent, the rate of volatilization slowed down, and the overall rate of natural attenuation began to decrease.

Figure 11b shows a comparison of TPH concentrations at different depths in the M-F-C tank. The TPH concentration in the upper layer of the M-F-C tank fluctuated greatly with time, and the “wick” phenomenon was obvious, while the TPH concentration in the lower media also fluctuated, but it was not obvious. Li [39] found that proper water saturation can strengthen the “wick” movement but that high water content made water occupy most of the pores, which hindered the migration path and inhibited the upward migration of vapor phase LNAPLs. This showed that dense media and high water content could block the migration of vapor phase LNAPL, so that the “wick” phenomenon was not obvious in fine sand media.

The TPH concentration in the middle fine sand layer decreased significantly over time (a high water content is conducive to the growth and reproduction of microorganisms and can promote the efficiency of microbial degradation of LNAPLs) showing that TPHs in the fine sand layer at a depth of 40 cm in the M-F-C tank decreased rapidly, which was due to the high water content accelerating the degradation rate. The LNAPL concentration in the lower layer of the M-F-C tank was not as high as that in the M-C-M tank. This was because the rising height of capillary water of coarse sand was lower than that of medium sand, so the lower point of the M-F-C tank was a long distance from the “oil slick” layer; thus, it was less affected.

#### 3.3.3. Natural Attenuation Process

In this study, TPH values at different points were monitored for six consecutive months from June to November. Figure 10b and Figure 11b show the fluctuation of TPH at different points with months. The natural attenuation rate of each point can be obtained by comparing the TPH values in November and June. The experimental results show that the attenuation rate of LNAPLs in different media was different (Table 5). The natural attenuation efficiency of diesel in fine sand was higher than that of coarse sand and medium sand. Affected by the “wick phenomenon”, the TPH of some points in the coarse sand and medium sand media fluctuated, resulting in a negative natural attenuation rate. The water content and lithology of the media were vital factors affecting the degradation efficiency of diesel. Under the same lithological conditions, the biological activity and biodegradation efficiency of diesel increased with increasing water content. Moreover, a finer soil media was more favourable for the adsorption of contaminants on the surface of soil particles and for the increase in biological activity, which was more favourable for its ability to degrade diesel. In our experiment, fine sand had the highest moisture content and thus the highest natural attenuation rate of diesel fuel in the fine sand media.

## 4. Conclusions

The main conclusions are as follows. (1) At the early stage of free infiltration, LNAPLs diffuse rapidly in a nearly circular shape in homogeneous media, and some migration front lines are irregular due to the heterogeneity of the microscopic soil media. The migration rate of LNAPLs in different lithologic media is significantly different, and the migration rate in coarse sand and medium sand media is much higher than that in fine sand media. (2) Precipitation is an important factor for the displacement of the free phase LNAPL and part of the residual LNAPL in the unsaturated zone. The removal rate in the coarse sand media is 40.78% and that in the fine sand media is approximately 10%. If the LNAPL contaminated site is coarse sand media, more LNAPLs enter the groundwater under the displacement of precipitation. (3) The natural attenuation of LNAPLs exists in both sand tanks of different media configurations. TPHs fluctuate in coarse sand and medium sand under the influence of the “wick phenomenon”, which is not obvious in fine sand; accordingly, TPHs in fine sand gradually decrease without much fluctuation. (4) The structure of the media plays a controlling role in the migration of LNAPLs in the vadose zone. First, the lithology and mutation interface of the media can affect the infiltration rate. Second, the displacement rate of LNAPLs in the vadose zone is also related to the lithology of the media under precipitation, and the displacement rate in fine sand is the lowest. Third, the natural attenuation rate of LNAPLs in the vadose zone is related to the lithology and water saturation of the media—the finer the particles are, the higher the natural attenuation rate. 

## Figures and Tables

**Table 1:** Soil particle size and specific gravity.

Type of Media	Particle Size Range (mm)	Specific Gravity (g/cm^3^)
Coarse Sand	0.5–2	2.62
Medium Sand	0.25–0.5	2.68
Fine Sand	0.05–0.25	2.74

**Table 2:** Part of the physical properties of diesel.

Type	Density (g/mL)	Dynamic Viscosity (cSt)	Surface Tension (dyne/cm)	Burning Point (°C)	Boiling Point (°C)
0#	0.829	2.54	27.8	220	180–410

No. 0 diesel is a kind of diesel. When the temperature of diesel vehicle is above 4 °C, 0# diesel is selected.

**Table 3:** Media configurations of the sand tank.

	M-C-M			M-F-C		
	Media	Filling Height (cm)	Porosity (%)	Media	Filling Height (cm)	Porosity (%)
Layer 1	Medium Sand	32	35.6	Medium Sand	33	39.7
Layer 2	Coarse Sand	29	32	Fine Sand	25	40.3
Layer 3	Medium Sand	33.5	36.2	Coarse sand	35	32

**Table 4:** Basic values of the experimental operation.

Configuration	Initial Water Table (cm)	Initial Capillary Fringe (cm)	Leakage Volume (mL)	Leakage Time (min)	Leakage Rate (mL/min)
M-C-M	5	20	3200	160	20
M-F-C	5	10	3200	160	20

**Table 5:** Natural attenuation rate of diesel in different soil media.

M-C-M	**Media**	**Medium Sand**		**Coarse Sand**			**Medium Sand**	
	Depth (cm)	20	30	40	50	60	70	80
	Natural attenuation rate (%)	37.33	29.21	10.32	10.73	53.00	54.41	0.59
M-F-C	**Media**	**Medium Sand**		**Fine Sand**			**Coarse Sand**	
	Depth (cm)	20	30	40	50	60	70	80
	Natural attenuation rate (%)	46.90	38.75	72.65	88.14	78.24	62.52	60.49

## Data Availability

The dataset used in this study are available from the corresponding author on reasonable request.
